# Degradation of Perovskite Thin Films and Solar Cells with Candle Soot C/Ag Electrode Exposed in a Control Ambient

**DOI:** 10.3390/nano11123463

**Published:** 2021-12-20

**Authors:** Mohammad Aminul Islam, Hamidreza Mohafez, Khan Sobayel, Sharifah Fatmadiana Wan Muhamad Hatta, Abul Kalam Mahmud Hasan, Mayeen Uddin Khandaker, Md. Akhtaruzzaman, Ghulam Muhammad, Nowshad Amin

**Affiliations:** 1 Department of Electrical Engineering, Faculty of Engineering, Universiti Malaya, Kuala Lumpur 50603, Malaysia; sh_fatmadiana@um.edu.my; 2 Department of Biomedical Engineering, Faculty of Engineering, Universiti Malaya, Kuala Lumpur 50603, Malaysia; 3 Solar Energy Research Institute, Universiti Kebangsaan Malaysia, Bangi 43600, Malaysia; sobayel@ukm.edu.my (K.S.); mahmud.1st@gmail.com (A.K.M.H.); akhtar@ukm.edu.my (M.A.); 4 Centre for Applied Physics and Radiation Technologies, School of Engineering and Technology, Sunway University, Petaling Jaya 47500, Malaysia; mayeenk@sunway.edu.my; 5 Graduate School of Pure and Applied Sciences, University of Tsukuba, Tsukuba, Ibaraki 305-8573, Japan; 6 Department of Computer Engineering, College of Computer and Information Sciences, King Saud University, Riyadh 51178, Saudi Arabia; ghulam@ksu.edu.sa; 7 College of Engineering, Universiti Tenaga Nasional (The National Energy University), Jalan IKRAM-UNITEN, Kajang 43000, Malaysia; nowshad@uniten.edu.my

**Keywords:** candle soot, C/Ag electrode, degradation rate, PbI_2_ conversion, defect distribution, perovskite solar cells

## Abstract

Perovskite solar cells (PSCs) have already achieved efficiencies of over 25%; however, their instability and degradation in the operational environment have prevented them from becoming commercially viable. Understanding the degradation mechanism, as well as improving the fabrication technique for achieving high-quality perovskite films, is crucial to overcoming these shortcomings. In this study, we investigated details in the changes of physical properties associated with the degradation and/or decomposition of perovskite films and solar cells using XRD, FESEM, EDX, UV-Vis, Hall-effect, and current-voltage (*I*-*V*) measurement techniques. The dissociation, as well as the intensity of perovskite peaks, have been observed as an impact of film degradation by humidity. The decomposition rate of perovskite film has been estimated from the structural and optical changes. The performance degradation of novel planner structure PSCs has been investigated in detail. The PSCs were fabricated in-room ambient using candle soot carbon and screen-printed Ag electrode. It was found that until the perovskite film decomposed by 30%, the film properties and cell efficiency remained stable.

## 1. Introduction

The organic-inorganic perovskite solar cells (PSCs) are considered a major recent discovery in the field of photovoltaics, which has attracted much attention recently. PSCs are giving an alternative approach that involves replacing the costly silicon-based solar cells [1,2,3,4,5]. Within a short period (around 10 years only), PSCs have already achieved a power conversion efficiency (PCE) of 25.8% [6], which exceeded the PCEs of most commercialized crystalline Si (21.2%), multi-crystalline Si (22.3%), CdTe based (22.1%), and copper-indium-gallium-selenide based (22.6%) thin-film solar cells [7]. However, since PSCs are susceptible to decay when exposed to humidity, oxygen, heat, light, and other factors, achieving long-term stability remains a substantial challenge. 

Particularly, the transition of perovskite films into their precursors is accelerated by water molecules, and the process is accelerated even more by light irradiation and/or heat and oxygen. This hydrolysis reaction of perovskite (Methylamine lead iodide, MAPbI_3_) starts when the material is exposed to a humid condition. From an optical point of view, the perovskite films have a dark brown color, which changed to yellow in degradation. So far, there have been very few reports [8,9] focusing on the influence of H_2_O, O_2_, and heat. Besides the perovskite films degradation via creating defective spots and cracking of columnar inter-grains, it has been reported that the characteristics of the carrier transport layers (such as ETL and HTL) play a critical role in bulk and interface degradation of PSC [10]. Kim et al. [11] proposed that the lifetime of PSC could increase via the incorporation of materials that had water-splitting capability within the adjacent layers (ETL or HTL) of the perovskite film. They engineered the HTL materials in which copper thiocyanate (CuSCN) was added to the poly (3-hexylthiophene) (P3HT) and found that the PSC maintained >9% of PCE even after a month stored in a water-saturated atmosphere. On the other hand, Guo et al. have been investigating the impact of light on the degradation of PSC operated under vacuum and a nitrogen ambient [12]. They reported that whereas light-induced phase segregation, morphological deformation, and lattice shrinkage occur in a vacuum, only lattice shrinkage occurs when solar cells are operated in a nitrogen ambient, resulting in improved device stability. Nonetheless, despite the scientific community’s best attempts to prevent the penetration of water molecules into the perovskite layers from the environment, device lifetimes, and stability remains yet to be critical, and certainly, novel disruptive approaches are necessary to move forward with PSC commercialization. It has been determined that as a long-term stability test, PSCs without encapsulation could be stable for over 500 h under 1.0 sun conditions and 1000 h under full sunlight [13]. So far, the longest reported stability for PSCs is approximately one year [14], which is certainly much shorter than the existing (approximately 25 years) commercialized PV technologies. 

For commercialization, PSCs must be able to operate without major degradation for almost 25 years in outdoor conditions. Last few years, the PSCs have been robustly engineered including change in the device structure, minimizing the number of active layers, and applying ancillary hydrophobic layers by which the lifetime of PSCs have been extended to several thousand hours [15]. Certainly, for achieving a highly stable device, more investigation into the degradation mechanism of perovskite materials is essential. In this work, we focused on the effect of humidity and heat for understanding the perovskite films, as well as PSC degradation rate. We are reporting the rate of conversion of perovskite thin films to PbI_2_ for three different samples, which have been kept in an ambient of relative humidity (RH) of 40% and a temperature of 30 °C. The prepared films have been placed in a humidity and temperature-controlled chamber and the change of the film’s crystalline, morphological, optical, and electronic properties have been observed for a different time duration ranging from 0 to 30 days. The complete PSCs with novel planner structures have been fabricated and their performance and stability have further been investigated in this study for similar environmental conditions and similar time duration.

## 2. Methodology

### 2.1. Preparation of Perovskite Films

**Figure 1:**
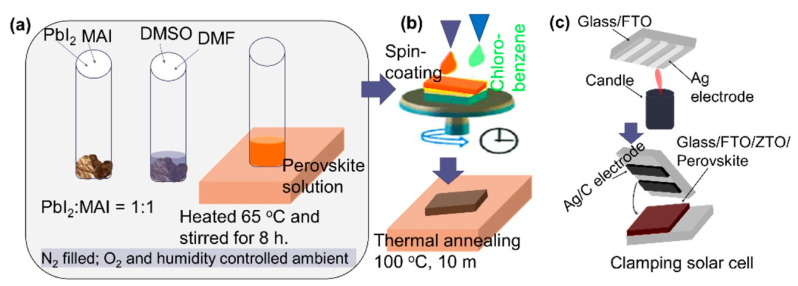
Schematic of the process flow for fabricating perovskite films and solar cells, (**a**) the preparation of perovskite precursor in an N_2_ and O_2_ controlled glove box, (**b**) perovskite film deposition via spin coating technique, and (**c**) back contacting via candle soot technique.

Commercial FTO-coated glass (Pilkington, Lathom, UK, 10 Ω/sq) substrates were cleaned, followed by sonication in a solution of detergent, deionized water, acetone, and isopropyl alcohol, and dried by N_2_ gas. The substrate was heated for several hours at around 60 °C, before depositing the perovskite films. A CH_3_NH_3_PbI_3_ perovskite precursor solution had been prepared by dissolving 461 mg PbI_2_ (99%, Sigma Aldrich, St. Louis, MO, USA) and 159 mg Methylammonium iodide (Dyesol Ltd. ASX:DYE, Queanbeyan, Australia) in 0.5 mL dimethylformamide (DMF) and 0.2 mL dimethyl sulfoxide (DMSO). The solution was prepared in an N_2_-filled glove box with O_2_ and an H_2_O content below 200 ppm and 1.0 ppm, respectively, and kept in the glove box for more than 8.0 h. For depositing the CH_3_NH_3_PbI_3_ perovskite layer, 60 μL of the aforementioned solution was put on the glass substrate and spin-coated for two steps: 10 s for 1000 RPM and 20 s for 3000 RPM. During spin-coating, 100 μL of the desired anti-solvent (chlorobenzene) was poured onto the spinning substrate after 5 s, while started the second step. The spin coating has been completed in the room ambient. Upon completion of the spin coating, a light-yellow colored perovskite layer was observed which became brown-black after the substrates were transferred to a hotplate set within the same atmospheric environment as the solution preparation. The samples were annealed at 100 °C for 10 min. The entire process was shown schematically in Figure 1a,b.

### 2.2. Preparation of Perovskite Solar Cells (PSCs)

For PSC fabrication, 100 nm of zinc-tin-oxide (ZTO) film had been deposited by sputtering on top of the FTO-coated glass substrate at 300 °C of substrate temperature. The properties of the ZTO thin films have been reported elsewhere [16]. The perovskite films were spin-coated on top of ZTO films, as mentioned above. The back contact layers had been employed by the candle soot and screen print method. However, although we modified the process, for details of the candle soot hole extracting layer and solar cell, readers are referred to the previous publication by Wei et al. [17]. In this study, the calcinated carbon electrode via candle soot was used since it was potentially a hole-extracting electrode material that was inexpensive, stable, environmentally friendly, and abundant. At first, the Ag metal electrodes were screen printed on top of the FTO coated glass substrate and dried at 100 °C for 20 m. Then, the “C” layer was deposited via candle soot technique on top of the Ag electrodes and finally, clamped with the FTO/ZTO/Perovskite stack for completing the solar cell. The process is schematically shown in Figure 1c.

### 2.3. Measurement and Characterization

The fabricated films and the solar cells had been placed in a humidity chamber with a constant temperature of 30 °C, 40% of relative humidity (RH), and under dark (light of 2–5 lumen). To obtain clear information about the degradation of the perovskite films, three different samples had been investigated together. It should be noted that a single sample was utilized for a single characterization only, and for subsequent characterizations, we used a similar sample rather than the same one. The XRD patterns were taken in the 2θ ranging from 10° to 50° using Cu Ka radiation wavelength of 1.5408 Å using ‘BRUKER aXS-D8 Advance Cu-Ka’ diffractometer (Billerica, MA, USA). The surface morphology, grain size, and grain growth were observed from the SEM images that were carried out using ‘LEO 1450 Vp’ (Carl Zeiss, München, Germany). The changes in carrier density, mobility, and resistivity were measured by the Hall-effect measurement tool ‘ECOPIA 3000′ (Bridge Technology, CO, USA). The optical properties were determined by UV-vis spectrometry using ‘Perkin Elmer Instruments Lambda35′ (Waltham, MA, USA). Furthermore, photoluminescence (PL) measurements of the films were carried out at room temperature by employing the “FLSP920 Edinburgh” spectrofluorometer (Fulton, MD, USA) using the exciting light wavelength of 458 nm. 3D FDTD optical simulations were utilized to investigate the optical losses that occurred as an impact of film decomposition. The performance of the cell with an area of 0.15 cm^2^ was evaluated under the illumination of 1.5 AM using ‘Gratings Inc. Solar Cell Tester: VI & power management system’ (Albuquerque, NM, USA).

## 3. Results and Discussion

**Figure 2:**
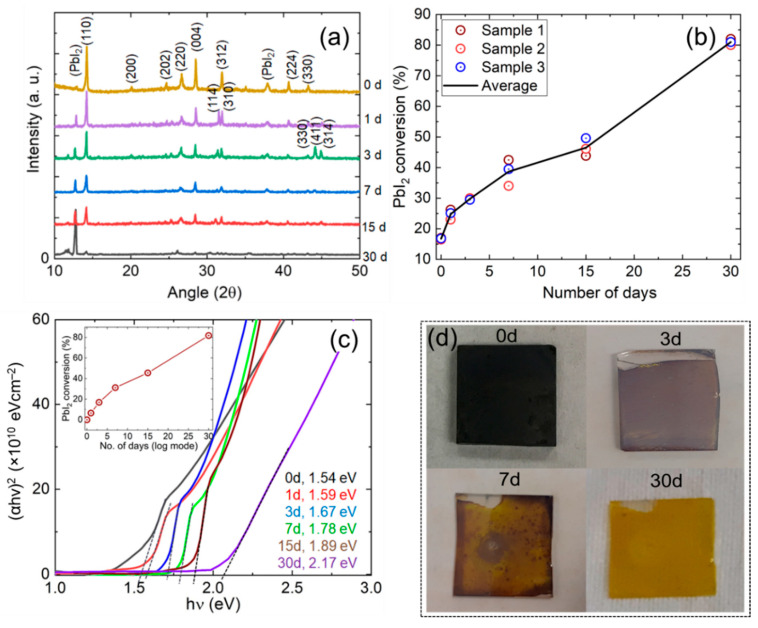
(**a**) XRD diffraction patterns of perovskite thin film (sample 1) exposed until 30 days, (**b**) estimated PbI_2_ conversion concerning the exposure time duration for three different samples, (**c**) optical bandgap evolution curves for perovskite films exposed for the different time duration, and (**d**) shows the pictorial view of perovskite films for the different number of days of exposure.

The time evolution of the perovskite films’ crystallographic properties that exposed for 30 days and sequentially observed the degradation are shown in Figure 2a. The diffraction peaks were found at 12.7°, 14.2°, 24.6°, 28.5°, 31.8°, 38.7°, 40.5°, and 44.0°, which corresponded to the crystal planes of (001) (for PbI_2_), (110), (202), (220), (310), (224), (134), and (404), respectively [18,19]. It was seen that the peak along with (312) plane dissociated to (114) and (310) after one day of exposure, and another two peaks along with (411) and (314) appeared after 3 days of exposure, indicating the decomposition of the perovskite film.

The change of crystallite size, including the peak height and full width half maximum (*β*) with the decomposition of perovskite thin film in the time range of 30 days, had also been estimated from the XRD patterns, as shown in Table 1. In this study, three different samples had been observed simultaneously for attaining a clear scenario of perovskite degradation, and the values of the parameters shown in Table 1 were the average values found from these three samples. The crystallite size (*D*) of the films were calculated using the following well-recognized formula. Further, to realize the atomic displacement, dislocation densities, and micro-strains had been estimated using the equation reported elsewhere [20]:

*D* = 0.89 *λ*/*β*cosθ (1)

*ε* = *β*/4tanθ (2)

*δ* = *n*/*D*^2^ (3)

where, *D* is the average crystallite size, *β* is the FWHM of the reflection peak having the same maximum intensity in the diffraction pattern, *λ* is the X-ray wavelength (0.15406 nm), θ is the Bragg diffraction angle, and *n* is a factor that is practically equal to unity for the lowest dislocation density.

**Figure 3:**
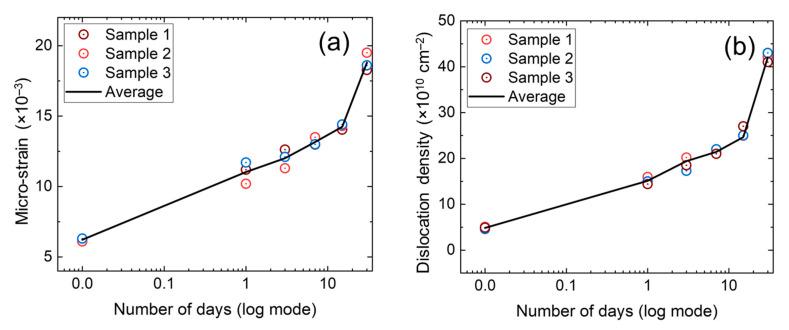
The change of (**a**) micro-strain and (**b**) dislocation density of the perovskite thin films exposed 30 days in 40% RH.

The changes in crystallite size, micro-strain, and dislocation density are shown in Table 1 and Figure 3a,b. It was found that peak height along the perovskite plane (110) was decreased with the increase of time. These phenomena indicated that the crystallographic perovskite structure was devouring due to moisture-induced decomposition. As the perovskite crystal decomposition led to the PbI_2_ conversion, it could be seen in Figure 2a that the PbI_2_ peak (001) was a rising alternative to the perovskite (110) peak. It was observed that the (110) plane peak position was slowly shifting towards a lower angle, indicating the increase of dislocation density and micro-strain. The increase of dislocation density and micro-strain, with the increase of exposed time, could also be realized from the change of mean crystallite sizes. It should be mentioned that Guo et al. [14] observed that perovskite degradation caused phase segregation, morphological deformation, and lattice shrinkage, which could contribute to crystallite size reduction. The decomposed perovskite to PbI_2_ may lead to point defects, interstitial defects, and/or vacancy into the perovskite lattice, which may influence the dislocation of atoms in the film.

Particularly, the lattice strain indicated that the atoms in the films were displaced from their reference-lattice positions. Alternatively, dislocation density points out the crystallographic imperfection of a film associated with the misregistry of the lattices, due to interstitial atoms and/or unlike vacancies in the film crystals. For understanding the micro-strain and dislocations developed in the film, PbI_2_ conversion had been estimated by employing the following equation:

PbI_2_ conversion (%) = [*I*_(PbI_2_)_/(*I*_(Perovskite)_ + *I*_(PbI_2_)_)] × 100 (4)

It could be seen from Figure 3a,b that the micro-strain and dislocation densities were proportionally increased with the increase of decomposed PbI_2_, as shown in Figure 2b. The proportional increase of micro-strains and dislocation densities with PbI_2_ conversion indicated that the perovskite decomposition process led to an increase of crystal defects, including point defects, edge dislocation, and precipitate. It was well known that the point defects led to lattice misfit and dislocation in the film structure [21].

Figure 2c shows the Tauc plots for extrapolating the bandgap of the perovskite films of 0 days to 30 days of exposure. The increase of bandgap with the increase in exposure time is seen in the figure. It is noteworthy that the transmittance of perovskite thin film was directly related to the PbI_2_ concentration in the film. The bandgap of the film increased from 1.54 eV to 2.17 eV after 30 days of exposure. Figure 2d shows the pictorial view of perovskite films for getting a clear indication of the impact of humidity on the film’s decomposition. The decomposition rate of perovskite films to PbI_2_ has been estimated using the simplified parabolic equation, considering band bowing (*b*) coefficient is zero:

(5) $$E_{g\left(AB\right)}=xE_{g\left(A\right)}+\left(1-x\right)\;E_{g\left(B\right)\;}\;i.e.,\;x=\left|\frac{E_{g\left(AB\right)-}E_{g\left(B\right)\;}}{E_{g\left(A\right)-}E_{g\left(B\right)\;}}\right|$$

where, $E_{g\left(AB\right)}$ is the bandgap of the exposed films, $E_{g\left(A\right)}\;$is the bandgap of the PbI_2_ (2.31 eV [22]), and $E_{g\left(B\right)}\;$is the bandgap of the perovskite film at 0 days of exposed. The estimated PbI_2_ conversion had been shown in the inset of Figure 2c, where it could be seen that the conversion until 1-day was slow, however it sharply increased by the increase of the exposure time and 80% conversion completed by 30 days of exposure. The conversion rate found from the UV-Vis spectroscopy study was quite similar to the findings in XRD.

**Figure 4:**
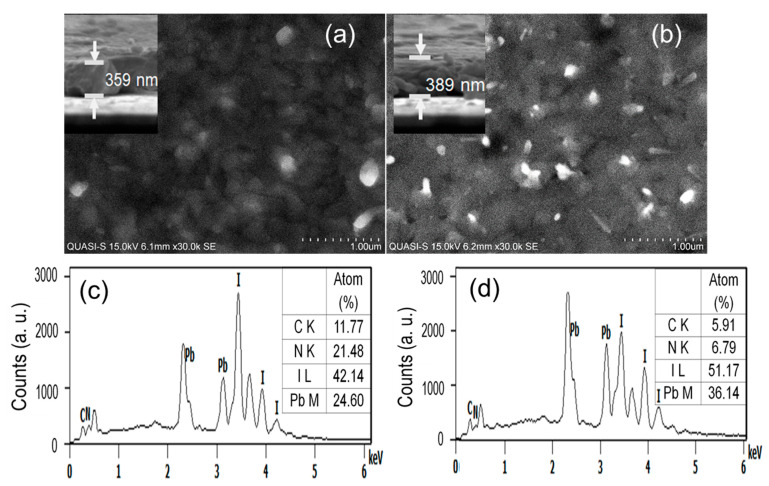
SEM images of (**a**) fresh and (**b**) 3 days exposed perovskite film (inset shows the SEM cross-section of the corresponding films), (**c**,**d**) elemental composition of the perovskite films corresponding to the SEM images.

Figure 4a,b showed the surface morphology of both the fresh and 3 days exposed film, respectively. Comparing the images of 0 days and 3 days, it could easily realize the moisture-induced morphological change in the film. The bright spots may be related to the high concentration of PbI_2_, which engendered under the moisture-induced decomposition. Figure 4c,d showed the EDX spectra for the corresponding films of 0 days and 3 days exposure. The increase of Pb and I was seen in the inset tables. Based on the findings, the decomposition of perovskite thin films could be predicted as follows [23]:

(6) $$CH_{3}NH_{3}PbI_{3}+H_{2}O\;↔\;CH_{3}NH_{3}PbI_{3}\cdot \;H_{2}O\;$$

(7) $$CH_{3}NH_{3}PbI_{3}\cdot H_{2}O\;↔CH_{3}NH_{3}I\;\left(aq.\right)+PbI_{2}\left(s\right)\;$$

(8) $$CH_{3}NH_{3}I\;\left(aq.\right)↔\;CH_{3}NH_{2}\;\left(aq.\right)+HI\left(aq\right)\;$$

(9) $$4HI\;\left(aq.\right)+O_{2}\;↔\;2I_{2}\left(s\right)+2H_{2}O$$

(10) $$2HI\;\left(aq.\right)\;↔H_{2}↑+I_{2}↑\;$$

**Figure 5:**
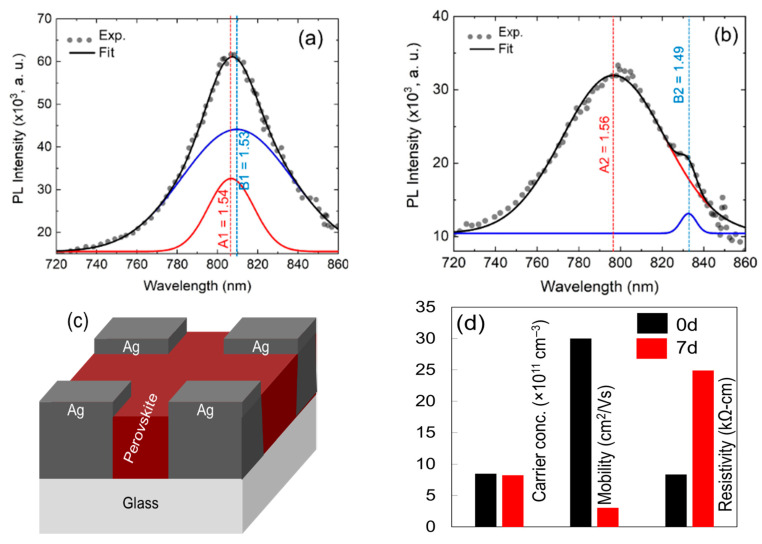
Photoluminescence spectra of perovskite films for (**a**) fresh and (**b**) 3 days of exposure (inset shows the estimated electronic recombination states for the films), (**c**) shows the schematic of prepared samples for Hall-effect measurement, and (**d**) electronic properties of perovskite films for two different exposing time, and (**b**) schematic of the prepared samples for Hall-effect measurement.

PL spectra as shown in Figure 5a,b have been studied to carry out the information regarding distinct energy states distributions in-between the conduction and valence bands, which are responsible for radiative recombination. The peak was fitted using a Gaussian fit, to assess the precise peak locations. The major peaks in the PL spectra were assigned by A1 and B1 for fresh perovskite, and A2 and B2 for 3 days exposed film, respectively. The most noticeable difference in Figure 5a,b is that the PL intensity dropped considerably after the third day of exposure. This decrease in PL intensity may have been due to the decrease of radiative recombination (alternatively, the increase of non-radiative recombination) via defect centers [24] that were created in perovskite during the decomposition process. Furthermore, the PL spectrum was broadened substantially, with a slight peak shift towards lower wavelengths for the 3 days exposed film. The PL broaden was thought to have arisen due to the exciton-phonon interaction and emission from the recombination of trapped charge carriers in the shallow and/or band edge level [25]. Furthermore, the peak shifting may have been related to the structural phase transition and/or film decomposition. Additionally, there was an emission peak at approximately 834 nm (1.49 eV) that may have been created by the recombination of the carrier from traps and/or surface states. These defects may have been associated with the PbI_2_ dissociation from perovskite, either in precipitate form or in the form of interstitial and antisites of Pb and I. This dominant trap position, either from the conduction band edge or valence band edge, was 0.07 eV for 3 days exposed film. The trap states were in good agreement with the trap state deduced by Sing et al. [26] and Samiee et al. [27], where they found that the trap states in perovskite films were located below the conduction band. Duan et al. also reported a trap state of 0.16 eV above the valance band, which associated with iodine interstitials (*I_i_*) [28]. In light of recent reports, among different native point defects, such as interstitials, vacancies, and antisites, *I_i_* only acted as a deep trap and non-radiative recombination center with low energy [28,29]. It had been reported that the perovskite film prepared under I-rich conditions, the deep trap with a formation energy of less than 0.2 eV, could be a form associated with the Pb_I_ antisite. Furthermore, lead-iodide complex anions could produce neutral antisite defects similar to the Pb_I_ [30]. Yin et al. [31] reported that deep transition levels were created owing to I atoms at MA sites (I_MA_), Pb interstitials (Pbi), Pb sites substitute by I (I_Pb_), and Pb atoms at I sites (Pb_I_) with reasonably high formation energies. is noteworthy that the defects with deep energy levels in the band gaps acted as Shockley-Read-Hall (SRH) non-radiative recombination centers, for which the minority carrier lifetime reduced, and consequently *Voc* was reduced. Similar deep level defects may have been created due to the PbI_2_ decomposition in this study, as was seen in the cell performance degradation shown in the next section.

The electronic properties have been carried out using the Hall-Effect measurement system for both fresh and 7 days exposed film. The prepared sample for the measurement is shown schematically in Figure 5c. Three different but similar films had been investigated, and the average values were depicted in Figure 5d. The Ag electrode had been employed in the four corners of the sample using quick-dry Ag past supplied by Ted Pella, Inc. (Redding, CA, USA) Fresh perovskite film had an average carrier concentration and mobility of 8.9 × 10^11^/cm^3^ and 31 cm^2^/V s, respectively. The findings were nearly identical to those of a prior investigation that was published elsewhere [32]. A significant change in mobility and resistivity was observed, however, change oppositely. The reduction of mobility indicated the increase of defects in the film. It could be easily assumed that the defects were generated due to the decomposition of perovskite to PbI_2_. However, the increase of resistivity was unknown; perhaps due to the decomposed PbI_2_ and absorbed water molecules influencing the resistivity of the film. We believe that the decrease in carrier concentration shown in Figure 5d may be due to a similar cause.

**Figure 6:**
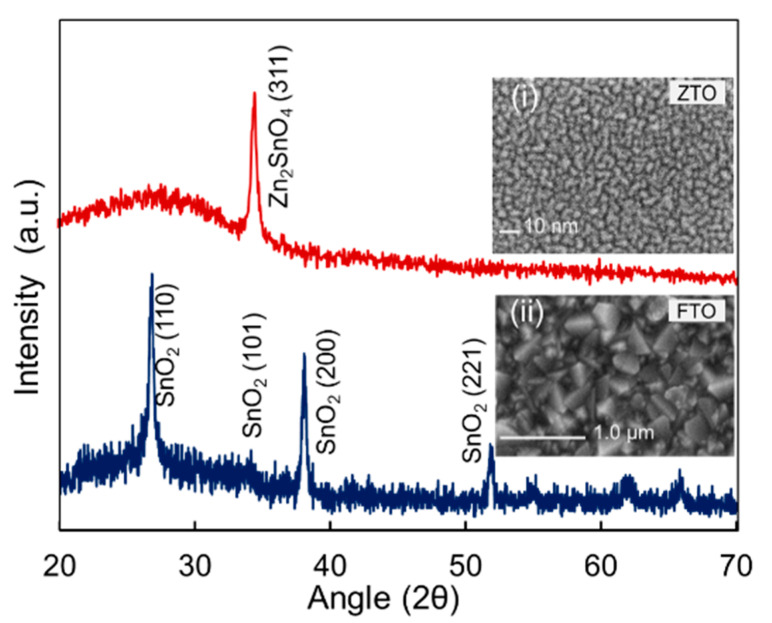
RD spectra of FTO and ZTO thin films used for fabrication of perovskite solar cells (inset shows the SEM image of (**i**) ZTO and (**ii**) FTO thin film).

**Figure 7:**
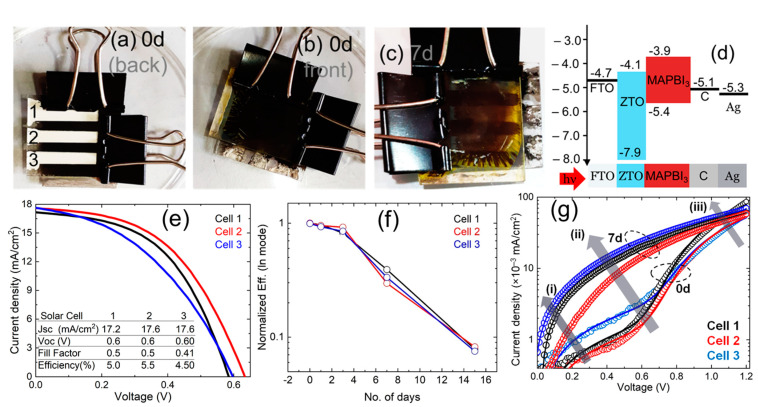
Pictorial view of perovskite solar cells; (**a**) from the backside of a fresh solar cell; (**b**) from the front side of the fresh solar cell, (**c**) from the front side of a 7 days exposed solar cell; and (**d**) schematic band diagram of the fabricated perovskite solar cells (**e**) *J*-*V* curves of three different solar cells as shown in Figure 7a, (**f**) the rate of degradation of conversion efficiency of the solar cell as exposed to moisture and (**g**) dark *I*-*V* characteristics of fresh and 7 days exposed perovskite solar cells (solid lines show the fitted curve for estimating the characteristic parameters and arrows as shown by (i), (ii), and (iii) are indicating the changes of the curves in the different regions).

The complete perovskite solar cell with novel structure had been primarily fabricated using FTO front contact, Zinc-tin-oxide (ZTO) as electron transport layer (ETL), and candle soot carbon nano-flex with Ag electrode. It was noteworthy that carbon electrodes had the advantage of working in PSCs without HTL and required no vacuum evaporation during device fabrication, lowering fabrication costs [33]. The structural properties and surface morphology of the FTO and ZTO are shown in Figure 6. For detailed properties of ZTO thin film, readers are referred to our previous publication [34]. Figure 7a,b show the pictorial view of the perovskite solar cells as in fresh condition; Figure 7c shows the pictorial view for 7 days of exposure and Figure 7d shows the schematic band diagram of the fabricated solar cells. The degradation of the PSC is visible in Figure 7c, where the Ag electrodes are visible through the diluted or decomposed perovskite thin film. We predicted that water molecules (H_2_O) from the ambient first diffused into the perovskite film and formed CH_3_NH_3_PbI_3_·H_2_O, which initiated slow degradation of the film and cell performance. Later migration of iodine ions induced a chain reaction and created a fast diffusion channel for H_2_O, O_2_, and other ions under heat and humid ambient. It should be noted that the perovskite grain boundaries and interface acted as the main pathway for ionic migration. Notably, numerous iodine ions started migrating from the hydrant area into the other non-affected areas and stimulating the hydration process, as shown in Equation (6) to Equation (10). Thus, the hydrated area expanded via the ion migration route and entered (or even through) the perovskite film forming a fast diffusion channel, and film properties were drastically degraded as discussed in the previous sections.

**Figure 8:**
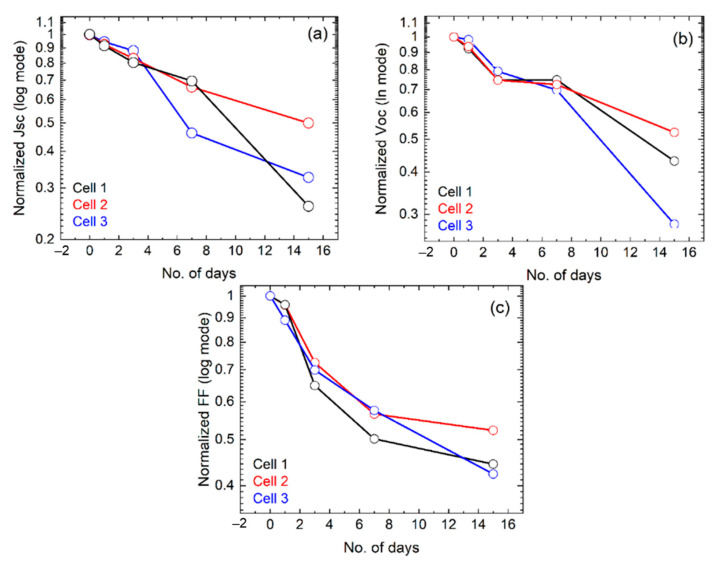
The degradation of (**a**) *Jsc*, (**b**) *Voc*, and (**c**) *FF* with respect to the exposure time.

Figure 7e shows the *J*-*V* curves of the fresh PSCs as shown in Figure 7a. It could be seen that the cell electrical parameters, including efficiency, were slightly different by their position in the substrate. The initial efficiencies were 5.5%, 5.2%, and 4.6% and had been found for cell 1, cell 2, and cell 3, respectively. The achieved efficiency was low which could be realized by observing the schematic band diagram, as shown in Figure 7d. It could be assumed that some of the optically generated electrons could move to the back contact, which was responsible for low electrical performance. It was evident that higher efficiency could be achieved in this structure, but essential to employ a suitable hole transport layer (HTL) as well as to optimize the candle soot process for the back contact (C/Ag) to create effective ohmic contact. It should be noted that we did not apply any HTL after the fabrication of perovskite films to prevent the effect of HTL on the degradation of perovskite films and/or solar cells. The variation of the normalized efficiency over the time of exposure is shown in Figure 7f. Furthermore, the variation of the normalized *Voc*, *Jsc*, and *FF* over the time of exposure could be found in Figure 8. It could be seen that cell 2, in which its position was in the middle of the substrate, was degraded somewhat slower than the other two. This may be due to the ionic migration process, as mentioned earlier. Besides, the efficiencies of PSCs were almost stable for 3 days and degraded sharply after such. It was found from XRD and UV-Vis that the conversion of PbI_2_ had occurred 30% in 3 days, thus we could conclude that the existence of 30% PbI_2_ in the perovskite film was tolerable for achieving a higher performance. Furthermore, after 15 days of exposure, the normalized PCE for all three cells remained below 10% of their initial values, as shown in Figure 7f. Estimating the degradation of *Voc*, *Jsc*, and *FF*, as shown in Figure 8, it was found that *FF* was reduced at a similar rate to the *Voc*, but *Jsc* seemed slightly more stable than *FF* and *Voc*. As mentioned previously, water molecules (H_2_O) from the ambient first diffused into the perovskite film and formed CH_3_NH_3_PbI_3_·H_2_O (Equation (6)), which initiated the slow degradation of *Jsc* [35]. The drastic degradation of *Voc* and *FF* due to the increase of recombination and/or decrease of a charge extraction efficiency of the device had been confirmed from the dark *I*-*V* analysis, as shown in Figure 7g. It had already been reported that the decrease of *V_OC_* in PSC was related to bulk and interface degradation after trap formation [36]. Particularly, the decomposition of perovskite films degraded the PSCs performance in three ways: (i) decrease of charge extraction efficiency due to the formation of space charge region by ion accumulation, (ii) defect redistribution (as seen in PL analysis) in the perovskite layer influence the carrier recombination [37] and (iii) the chemical reaction with the selective layers (ETL and HTL), even with the electrode [38] which could be seen in Figure 7c. It is noteworthy that all the above facts could reduce the shunt resistance and increase the series resistance as seen in Table 2, which in turn reduced the *FF* and *Voc*. Furthermore, the above facts could reduce the cell’s junction quality, in which it became less capable of separating electrons and holes [39]. Overall, the *Voc* of solar cells was directly related to the carrier lifetime that reduced, due to the increase or redistribution of recombination center and/or defects via decomposition of perovskite film.

The diode characteristics were retrieved by fitting the dark *I*-*V* curve into a single diode model to provide a clearer picture of the changes in the PSCs’ electrical properties, as shown in Figure 7g. The saturation current (*I*_0_), shunt resistance (*R*_sh_), series resistance (*R*_s_), and ideality factor (*n*) were determined using the following Equation [34]:

(11) $$I\left(V\right)=I_{0}\left[\exp\left(\frac{q\left(V-JR_{s}\right)}{nkT}\right)-1\right]+\frac{V-IR_{s}}{R_{\mathrm{sh}}}$$

where *q* is an elementary charge, *k* is Boltzmann’s constant, and *T* is the absolute temperature (300 K). *I*_0_, *R*_sh_, and *R*_s_ were positive, and *n* was limited to be greater than one. In Figure 7g, the arrow signs in the region (i) indicated the reduction of shunt resistance, in the region (ii) showed the increase of saturation current density, and region (iii) showed the increase of series resistance in cell 1, cell 2, and cell 3 for exposing 7 days in a humid ambient. The change in the ideality factor, saturation current density, series, and shunt resistance extrapolated from the dark *I*-*V* is shown in Table 2. Table 2 shows a clear scenario of cell electrical properties deterioration. Further, the high ideality factor, even in the fresh solar cell, indicated the low conversion efficiency and *FF* as seen in light *I*-*V* characteristics. In this case, we are suspecting that the additional SRH recombination centers had been created as a cause of iodine and Pb ions migration into the perovskite crystal and/or perovskite/ZTO or perovskite/C interface led to increase surface and bulk recombination and altered the device ohmic properties. As shown in Table 2, the saturation current density (*I*_0_) was increased significantly after 7 days of exposure, indicating that significant recombination occurred via defect levels where many defects existed within the depletion region of the exposed cell. The high defect density in the deflation region was a cause for lowering *Voc* and *FF*. The increase of series resistance was observed after 7 days of exposure, which could be related to the decrease of charge transfer capability of ZTO ETL as an impact of perovskite decomposition [38] and impacted grain boundary of perovskite films. In contrast, the cell lost almost complete photovoltaic characteristics after 30 days of exposure.

## 4. Conclusions

The degradation of perovskite films and solar cells has been studied at 40% RH, 30 °C ambient temperature, and 2 to 5 lumens of light. The decomposition rate of perovskite film to PbI_2_ has been estimated from XRD and UV-Vis spectroscopy. It has been observed that the perovskite peak (110) has been shifted to a lower diffraction angle, and the dissociation of peak (312) and (411) indicate an increase of dislocation and micro-strain in the perovskite structure. PL spectra show that the defect levels are redistributed as an impact of perovskite decomposition. The surface morphology and electronic properties, such as mobility, carrier concentration, and resistivity have also been altered significantly due to the perovskite decomposition. This decrease in PL intensity for exposed films indicates a decrease in radiative recombination and an increase in defect centres produced in perovskite films as a result of decomposition. Furthermore, the PL broaden was observed, which was thought to arise due to emission from the recombination of trapped charge carriers in the shallow and/or band edge level. The complete solar cells have been fabricated in-room ambient using candle soot carbon with screen printed Ag electrode, and the highest conversion efficiency of 5.5% has been achieved so far. It has been found that the decomposition primarily occurred slowly, however, exponential increment takes place after the 30% of PbI_2_ conversion. Based on the analysis of PbI_2_ conversion and efficiency loss, it was found that up to 30% PbI_2_ conversion is tolerable for maintaining higher efficiency. Dark I-V characteristics show that the cell shunt resistances are reduced and alternatively, the saturation current densities are increased as an impact of defect redistribution in the perovskite layer via decomposition. Cell *I*-*V* characteristics show that the cell near the edge is affected much more than the cell in the middle by humidity and temperature.

## Figures and Tables

**Table 1:** The extrapolated average peak height, FWHM, and crystallite size of the perovskite films were exposed for 30 days.

Time	Angle, 2θ (Degree)	Average Peak Height, *h* (a. u.)	Average FWHM, *β* (×10^−3^ Rad.)	Average Crystallite Size, *D* (nm)
0 days	14.26	586	3.14	44.50
1 day	14.21	441	5.58	25.03
3 days	14.18	348	6.28	22.25
7 days	14.18	228	6.45	21.65
15 days	14.17	214	6.97	20.02
30 days	14.15	117	9.07	15.40

**Table 2:** Electrical characteristics of PSC extracted from dark *I*-*V* curves.

		*n*	Absolute Deviation (%)	*I*_0_ (A)	Absolute Deviation (%)	*Series Resistance* (*R*_s_) (Ω)	*Shunt Resistance* (*R*_sh_) (Ω)
Fresh	Cell 1	2.16	0.025	5.56 × 10^−8^	2.51 × 10^−3^	68.9	280.5
	Cell 2	2.14	0.027	3.65 × 10^−8^	2.11 × 10^−3^	65.1	450.2
	Cell 3	2.49	0.021	2.25 × 10^−7^	2.16 × 10^−3^	70.4	186.5
7 days	Cell 1	2.74	0.026	5.45 × 10^−6^	2.88 × 10^−3^	132.5	118.6
	Cell 2	2.64	0.023	7.04 × 10^−7^	2.59 × 10^−3^	140.2	351.3
	Cell 3	2.56	0.021	2.81 × 10^−6^	2.94 × 10^−3^	128.7	151.5

## Data Availability

The data that support the findings of this study are available from the corresponding author upon reasonable request.
